# Preparation of a mimetic and degradable poly(ethylene glycol) by a non-eutectic mixture of organocatalysts (NEMO) *via* a one-pot two-step process†‡

**DOI:** 10.1039/c9ra09781c

**Published:** 2019-12-04

**Authors:** S. Moins, P. Loyer, J. Odent, O. Coulembier

**Affiliations:** Laboratory of Polymeric and Composite Materials, Center of Innovation and Research in Materials and Polymers (CIRMAP), University of Mons (UMons) Place du Parc 23 7000 Mons Belgium olivier.coulembier@umons.ac.be; Inserm, INRA, Univ Rennes, Institut NUMECAN (Nutrition Metabolisms and Cancer) UMR-A 1341 UMR S 1241 F-35000 Rennes France

## Abstract

A one-pot, two-step method for the preparation of degradable PEG is here presented. The full process addresses the requirements imposed by green chemistry and involves the use of a single and nontoxic non-eutectic mixture of organocatalysts. The strategy relies on the polycondensation of PEG800 after its functionalization by bio-derived 5-membered γ-butyrolactone.

## Abstract 2

A one-pot, two-step method for the preparation of degradable PEG is here presented.

## Graphical abstract

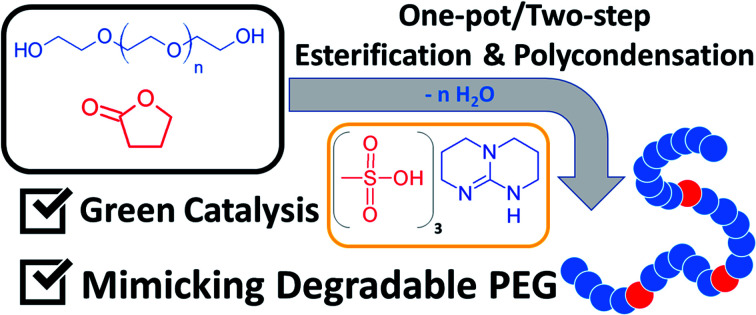

Nature is replete with examples of processes that have been perfected over the ages and which are now tentatively replicated by scientists using various chemical tools. Among them, PEGylation represents a successful approach in drug delivery by enhancing the potential of peptides and proteins as therapeutic agents.^1^ More specifically, PEGylation involves the modification of a protein, polypeptide, DNA and RNA as well as small molecules by the linking of polyethylene glycol (PEG) chains. The effects of surface coverage by the hydrophilic, biocompatible and FDA-approved PEG are mainly characterized by non-recognition by the immune system and a reduced tendency of nanoparticles to aggregate by steric stabilization.^2^ A disadvantage of PEG is its non-degradability. If low-molar-mass PEGs are preferable, oligomers with a molar mass below 400 Da are also toxic in humans.^3^ Contradictorily, to allow a complete excretion of the polymer, the molar mass should not exceed the renal clearance threshold of 40–60 kDa.^1^ As for other polymers, PEGs are excreted by urines or faeces but high molar-mass PEGs accumulate in the liver causing a macromolecular syndrome.^2^ To get rid of such a problem, efforts have been devoted on the preparation of degradable PEG by incorporating degradable segments/linkages in the main backbone. Two possible routes allow degradable PEG to be prepared: (i) the ring-opening copolymerization (ROcP) of ethylene oxide and another heterocyclic monomer and (ii) the polycondensation of (oligo)ethylene glycol in presence of adequate and functionalized synthons. ROcP presents the advantage to perfectly control both molar mass and dispersity of PEG derivatives but suffers from a non-uniformity of degradable segment distribution through the main backbone. Comparatively, PEG polycondensates exhibit broad dispersity values but are defined by a relatively homogenous repartition and a large choice of degradable linkages including esters,^4^ disulfide,^5^ acetal,^6^ imine^7^ and carbonate^8^ functions. Another problem for the preparation of PEG polycondensates relates to the use of potentially toxic chemicals and the release of dangerous gases and sub-products produced either during the end-functionalization of the (oligo)ethylene glycol or the polycondensation process.

In the present contribution, we provide a complete description of PEG polycondensates synthesis in solvent-free condition respecting the workable definition of green chemistry. In substance, a one-pot/two-step process has been applied to both end-functionalize an oligoethylene glycol synthon and induce its polycondensation to obtain a mimetic and degradable PEG. Such a process involves the use of a Non-Eutectic Mixture of Organocatalysts (NEMO), is free of waste and avoid the use and production of toxic and/or hazardous reagents and solvents.

Due to its low strain energy,^9^ the bio-derived five-membered γ-butyrolactone (γ-BL) is referred as a “non-polymerizable” monomer. At the exception of reactions realized under cryogenic conditions,^10^ the ring-opening polymerization (ROP) of γ-BL is limited to low oligomerization under ambient pressure and high temperature due to its high equilibrium monomer concentration.^11^ Such incapability of polymerizing has been put to good use to end-functionalize an oligoethylene glycol (MW 800 g mol^−1^, PEG800) by few units of γ-BL. After screening different reaction conditions, we selected a non-stoichiometric complex of methanesulfonic acid (MSA) and 1,5,7-triazabicyclo[4.4.0]dec-5-ene (TBD) (3 : 1) to catalyse the process at 130 °C. The interest of such Non-Eutectic Mixture of Organocatalysts (NEMO) relies on its ability to conduct bulk polycondensation of a variety of diols,^12^ offering then the possibility to both functionalize and polycondensate the PEG800 in a one-pot process (Scheme 1).

**Scheme 1:**
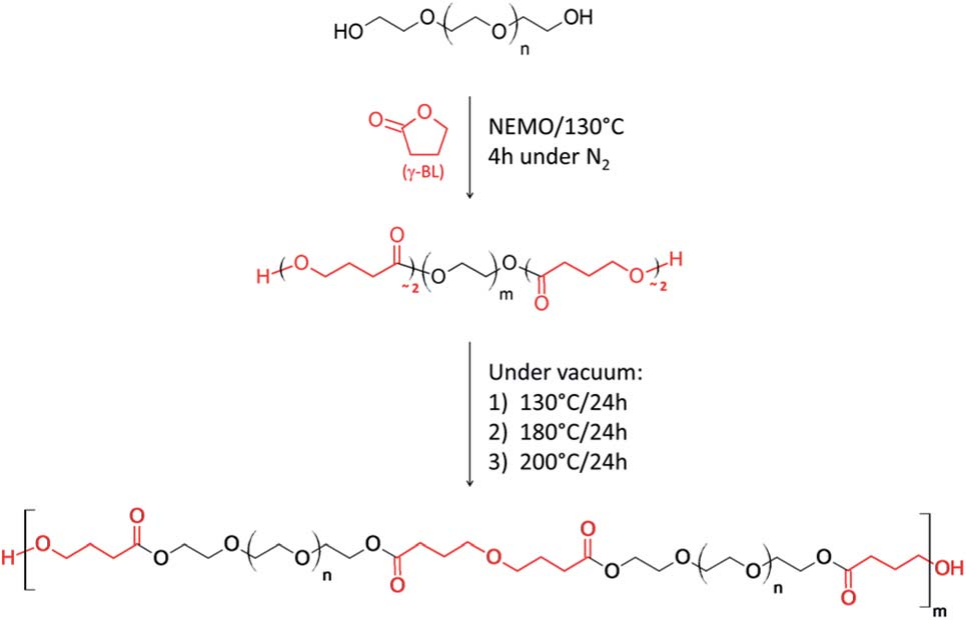
Expected end-functionalization of PEG800 and polycondensation one-pot/two-step process catalyzed by NEMO.

Prior to any polycondensation reaction, the functionalization of PEG800 was studied and optimized. After screening different conditions, it appeared that the optimum conditions of functionalization in terms of time and efficiency of reaction necessitate to use a 10-fold excess of γ-BL as compared to the PEG800 and an amount of catalyst of 0.2 molar equivalent ([PEG]_0_/[γ-BL]_0_/[NEMO]_0_ = 1/10/0.2). Under such conditions, and as attested by ^1^H NMR analysis, 52% of the PEG800 hydroxyl end-groups were functionalized by ∼1.5 units of γ-BL after 4 hours. Increasing the reaction time to 6 hours does not allow to improve that conversion. To respond to the challenges addressed by the green chemistry in terms of waste, a simple vacuum treatment of the medium (prior to the polycondensation reaction) allows the excess of unused γ-BL to be recycled and reused for another reactions. Fig. 1 presents the ^1^H NMR analyses of the PEG800 after reaction with excess of γ-BL and treated under vacuum (Fig. 1A) as well as the product of the evaporation (Fig. 1B). As compared to the ^1^H NMR analysis of the γ-BL, the recovered and unreacted monomer proved chemically pure and reusable.

**Fig. 1:**
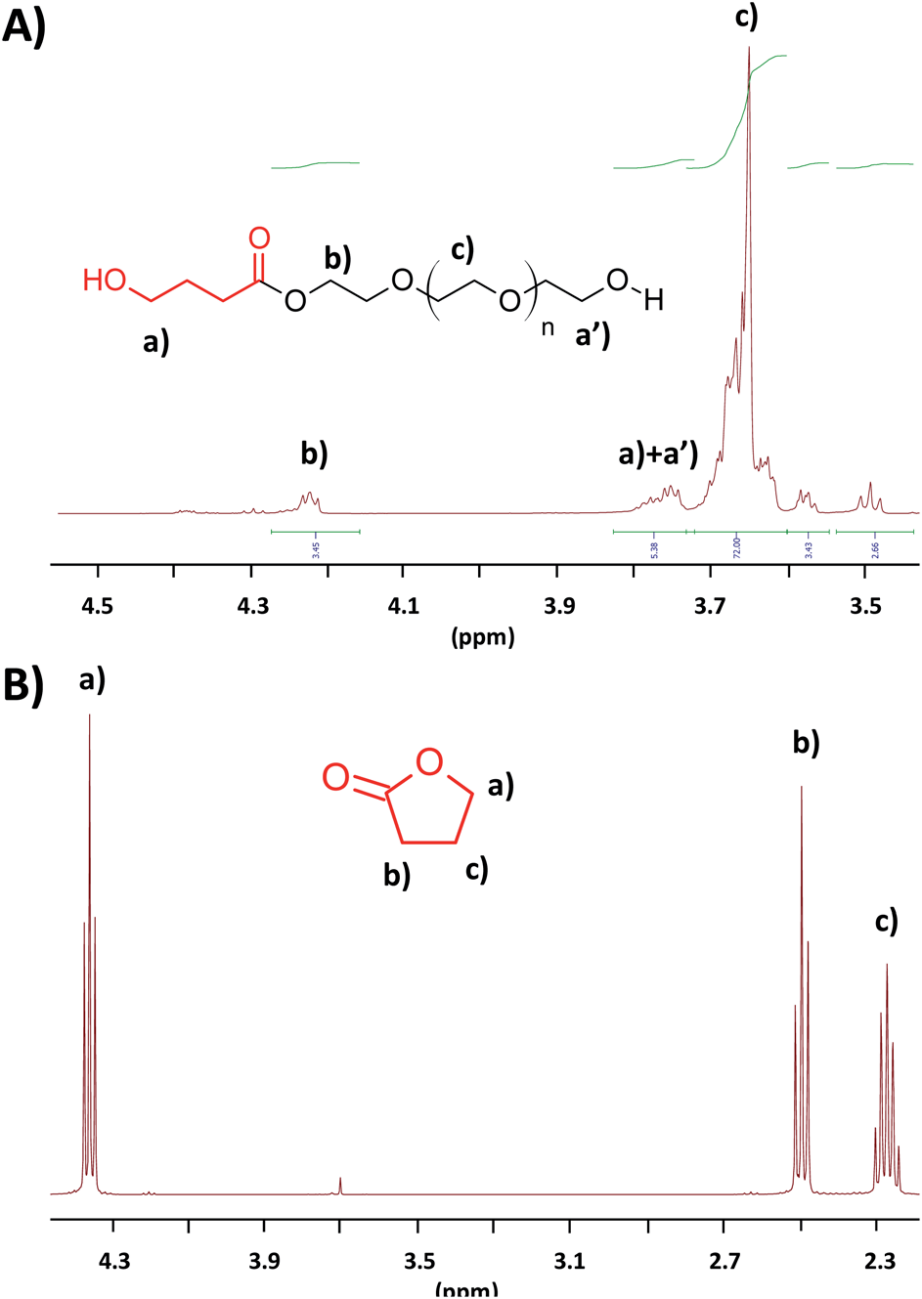
^1^H NMR analyses (in CDCl_3_, r.t.) of the PEG800 after end-functionalization and vacuum treatment (A) and of the product of evaporation (B).

Recently some of us reported on the bulk self-condensation of aliphatic diols as a route to polyether homo- and copolymers.^12^ Following the same strategy, the polycondensation of the end-functionalized PEG800 was realized by taking advantage of the NEMO already present in the macromonomer. Briefly, the medium was kept under vacuum and was the object of successive thermal treatments starting at 130 °C for 24 h, 180 °C for 24 h and finishing by a final step at 200 °C for 24 h. Note here that during the first thermal treatment the excess of γ-BL was recovered and reused for another reaction. The polycondensation was monitored using ^1^H NMR spectroscopy by following the disappearance of the hydroxymethylene protons present between *δ* 3.72 ppm and *δ* 3.80 ppm. Fig. 2 presents the comparison between the end-functionalized PEG800 before and after the three thermal treatments. As expected, the hydroxymethylene protons disappeared to the benefit of a narrower polyether signal at *δ* 3.64 ppm while butyryl ester groups are still present (here represented by the methylene oxycarbonyl protons at *δ* 4.22 ppm).

**Fig. 2:**
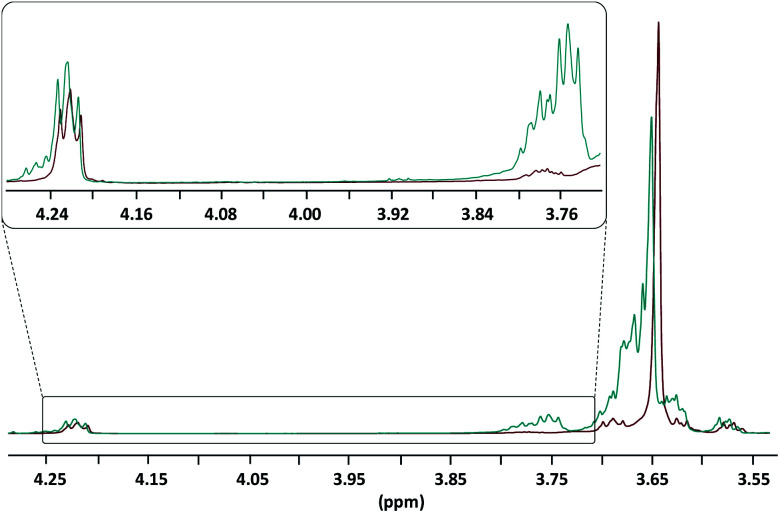
^1^H NMR analyses (in CDCl_3_, r.t., zoomed between *δ* 3.55 ppm and *δ* 4.30 ppm) of the end-functionalized PEG800 before (blue spectrum) and after thermal treatments (red spectrum).

SEC analyses of the polycondensates obtained after each thermal treatment show unimodal traces with dispersity values close to 2.0 and *M*_p,SEC_ increasing gradually in function of the thermal treatment applied (Fig. 3). In a view of future applications as micelle-forming copolymers, the molar mass of the prepared mimicking PEG was limited to 17 K g mol^−1^ knowing that most of the PEG hydrophilic block involved in biomedical micelles are comprised between 1 and 15 kDa.^13^

**Fig. 3:**
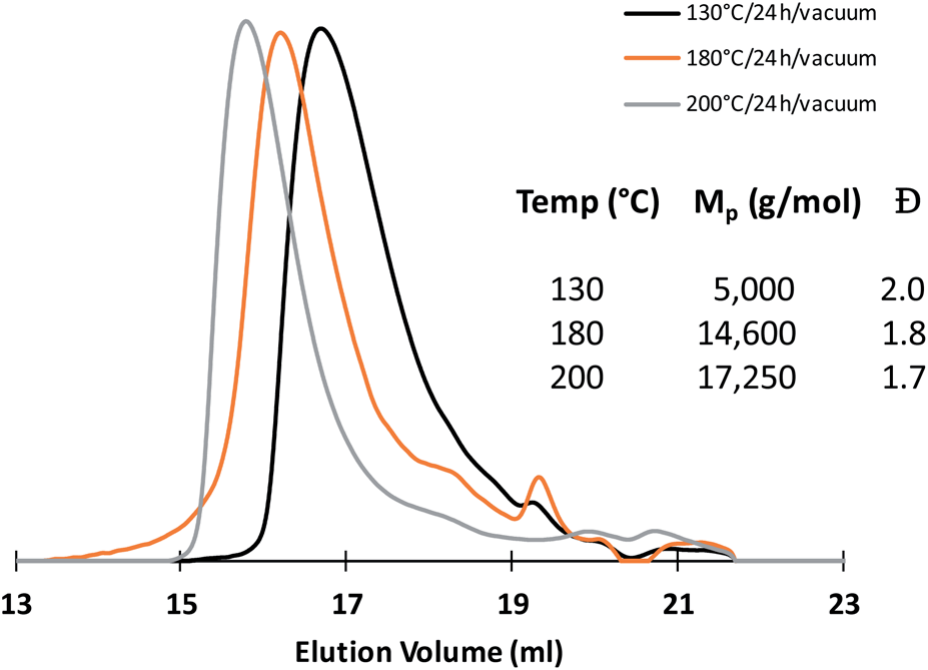
SEC chromatograms recorded after each thermal treatment of the polycondensation process.

Lastly, the degradation of the mimicking poly(ethylene glycol) polycondensate^14^ (*M*_p,SEC_ = 17 250 g mol^−1^) was performed by hydrolysis of the ester linkages in a 0.5 M NaOH solution. After 15 hours under stirring at r.t., the solution was lyophilized, and the recovered product analysed by SEC and ^1^H NMR analyses.

The ^1^H NMR analysis attests on the total degradation of the ester linkages as exemplified by the disappearance of the methylene oxycarbonyl signal initially present at *δ* 4.22 ppm and the reappearance at *δ* 3.72 ppm of the hydroxy methylene end-group protons of the PEG (Fig. 4). SEC analysis also concludes on a total degradation of the mimicking PEG by demonstrating that the as-obtained degraded product presents a similar chromatogram to the one of the PEG800 used before NEMO treatment (Fig. S1, ESI‡).

**Fig. 4:**
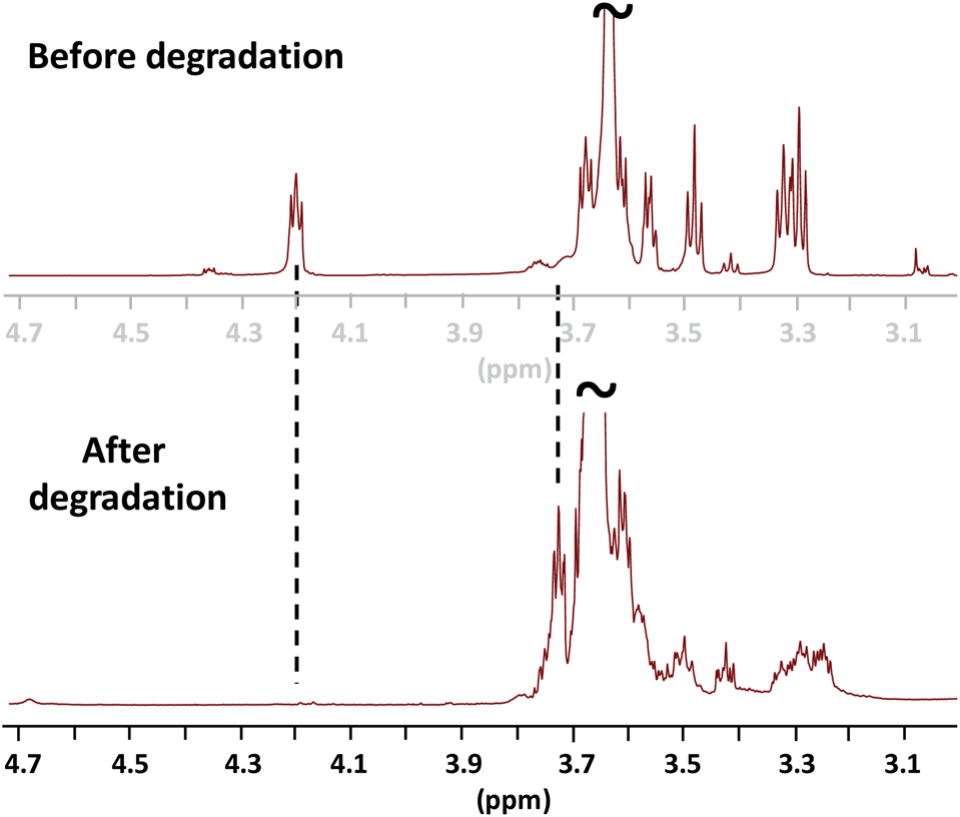
^1^H NMR analyses (in CDCl_3_, r.t., zoomed between *δ* 3.0 and *δ* 4.7 ppm) before (top) and after degradation of the mimicking PEG, *M*_p,SEC_ = 17 250 g mol^−1^ (bottom).

When considering toxicity, the biocompatibility of the initial polymer, the degradation products and the remaining catalyst must be taken into account. Literature data indicate that PEG does not present unquantified risk to humans^15^ and P(γ-BL) easily degrades to give γ-hydroxybutyric acid, a naturally occurring metabolite in the body.^16^ As NEMO catalyst remained in our mimicking PEG (∼5.5 mol% after one precipitation), we then found crucial to study its putative cytotoxicity *in vitro* (Fig. 5). The effect of NEMO catalyst on cell viability was first studied using the differentiated human hepatocyte-like HepaRG cells (Fig. 5A), which express most of the liver specific enzymes involved in xenobiotic metabolism^17^ allowing to assess hepatic toxicity of synthetic compounds.^18^ The NEMO catalyst was added to culture media at concentrations ranging from 0.065 to 13 mM and the cell viability was assayed by measuring the intracellular ATP content. The NEMO catalyst did not affect cell viability up to 1.3 mM while at 3.2 and 6.5 mM the ATP content was significantly decreased with an IC_50_ at 8.53 ± 0.24 mM. At the highest concentration of 13 mM, the ATP content was below the detection level demonstrating that only high concentrations of compound were cytotoxic. The toxicity of the NEMO catalyst was also evaluated with progenitor HepaRG cells (Fig. 5B) that actively proliferate but are undifferentiated. The NEMO catalyst also induced a strong cytotoxicity only at the high concentrations of 6.5 and 13 mM. To determine the mechanism of toxicity, we first studied the plasma cell membrane integrity of progenitor HepaRG cells using the trypan blue stain (ESI 1‡). Less than 15 minutes after incubating the cells with medium containing 13 mM of NEMO catalyst, the integrity of plasma cell membrane was lost correlating with a pH value at 2.91 (Fig. 5C and ESI 1‡). To prevent the pH decrease in culture medium, 100 mM Hepes buffer were added to culture medium that maintained the pH at 7 (Fig. 5C). In this condition, the NEMO catalyst did not significantly affect the ATP content even at the highest concentration of 13 mM.

**Fig. 5:**
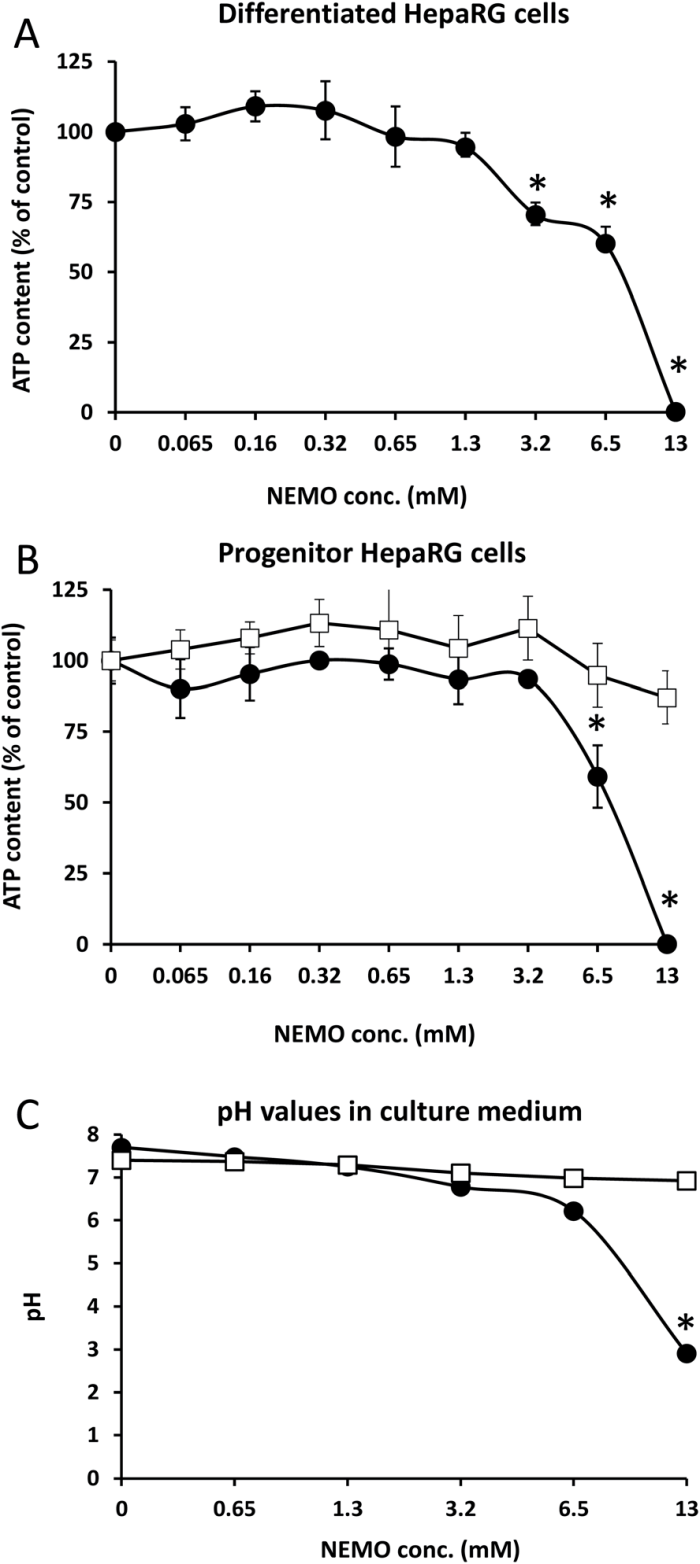
Determination of the relative ATP contents in differentiated (A) and progenitor (B) HepaRG cells [arbitrarily set at 100% in untreated cultures] and (C) pH in culture media at different concentrations of NEMO catalyst [expressed in mM]. Media without Hepes buffer (dark circles) and media supplemented with 100 mM Hepes buffer (white squares). Statistics: Student *t* test with *p < 0.01.

## Conclusions

Respecting the Green Chemistry guidelines, a one-pot/two-step process method to produce mimicking and degradable PEG was elaborated. Both functionalization and polycondensation of a commercially available PEG800 were carried out using a non-eutectic mixture of MSA and TBD organocatalysts, also called NEMO. NEMO was found not cytotoxic *per se* but produced an acidic pH when added in culture medium at very high concentrations. The non-polymerizable bio-derived 5-membered γ-BL appeared pertinent for an easy end-functionalization of the oligo-PEG800 in presence of 0.2 mol of NEMO at 130 °C for 4 hours under nitrogen. Under vacuum, the excess of γ-BL was easily recovered and reused, while the thermal/vacuum treatment of the medium promoted the polycondensation of the end-functionalized PEG to lead to the production of biomimicking PEG of *M*_*n*_ > 17 000 g mol^−1^. Finally, the degradation of the polycondensate PEG was performed by hydrolysis in a 0.5 M NaOH solution. After 15 hours, SEC attested on the total degradation of the PEG, demonstrating its mimicking behavior.

## Conflicts of interest

There are no conflicts to declare.

## Supplementary Material

RA-009-C9RA09781C-s001
