# Supplementary material for: Preparation of a mimetic and degradable poly(ethylene glycol) by a non-eutectic mixture of organocatalysts (NEMO) via a one-pot two-step process
Source: RSC Adv. 2019 Dec 4;9(68):40013–6. doi: 10.1039/c9ra09781c (PMC9076182; doi:10.1039/c9ra09781c)
Supplement: RA-009-C9RA09781C-s001 [file RA-009-C9RA09781C-s001.pdf]

Electronic Supplementary Material (ESI) for RSC Advances.
This journal is © The Royal Society of Chemistry 2019

SUPPORTING INFORMATION

Preparation of a Mimetic and Degradable Poly(ethylene glycol) by a Non-Eutectic Mixture of Organocatalysts (NEMO) One-Pot Two-Step Process

S. Moins[a], P. Loyer[b], J. Odent[a] and O. Coulembier[a*]

a) Laboratory of Polymeric and Composite Materials, Center of Innovation and Research in Materials and Polymers (CIRMAP), University of Mons (UMons), Place du Parc 23, 7000 Mons, Belgium
b) Inserm, INRA, Univ. Rennes, Institut NUMECAN (Nutrition Metabolism and Cancer) UMR-A 1341, UMR S 1241, F-35000 Rennes, France

1. EXPERIMENTAL PART

1.1 MATERIALS

Oligoethylene glycol (MW 800 g.mol^{-1}, PEG800, Sigma-Aldrich) was used as received after being dried by three successive azeotropic distillations by addition of toluene. γ-Butyrolactone (γ-BL, Acros Organics) was dried over CaH_2 for 24 hours and distillated under vacuum. 1,5,7-Triazabicyclo[4.4.0]dec-5-ene (TBD, 98%, Aldrich) was dried at 80°C overnight under vacuum. Methanesulfonic acid (MSA, 99%, Sigma-Aldrich) was used as received. Chemicals were stored and manipulated in a glovebox (H_2O < 3ppm, O_2 < 1ppm)

1.2 METHODS

Preparation of the NEMO catalyst

In a glovebox and in a previously dried Schlenk-type glassware, 0.1g of methanesulfonic acid (MSA, n = 1.11 x 10^{-3} mol) were added to 0.05g of 1,5,7-triazabicyclo[4.4.0]dec-5-ene (TBD, n = 3.72 x 10^{-4} mol). Out of the glovebox, the inhomogeneous mixture was thermally treated at 90 °C for 30 minutes to get a transparent and homogenous solution.

Polycondensation of end-functionalized PEG800

In a glovebox and in a previously dried Schlenk-type glassware equipped with a stirring bar, 1g of PEG800 (n = 1.25 x 10^{-3} mol) was added to 1.075g of γ-BL (10 eq., n = 1.25 x 10^{-2} mol). After homogenization, 0.107g of NEMO (0.2 eq., n = 2.5 x 10^{-4} mol) were then introduced. Out of the glovebox, the sealed reaction vessel was submerged into a pre-heated oil bath at 130 °C. After 4 hours, the reaction medium was kept under vacuum for one day at the same temperature. After such period of time, the reaction vessel was heated up to 180 °C (for 24 hours) and 200 °C (for 24 hours), still under vacuum. The reaction was stopped under the air by cooling the vessel under cold water. The sample was dissolved in a minimum of chloroform, precipitated in cold ether, filtered and dried under vacuum for 12h at r.t.

Impact of NEMO catalyst on pH in culture medium and alteration of plasma cell membrane by acidification in the human hepatoma HepaRG cells.

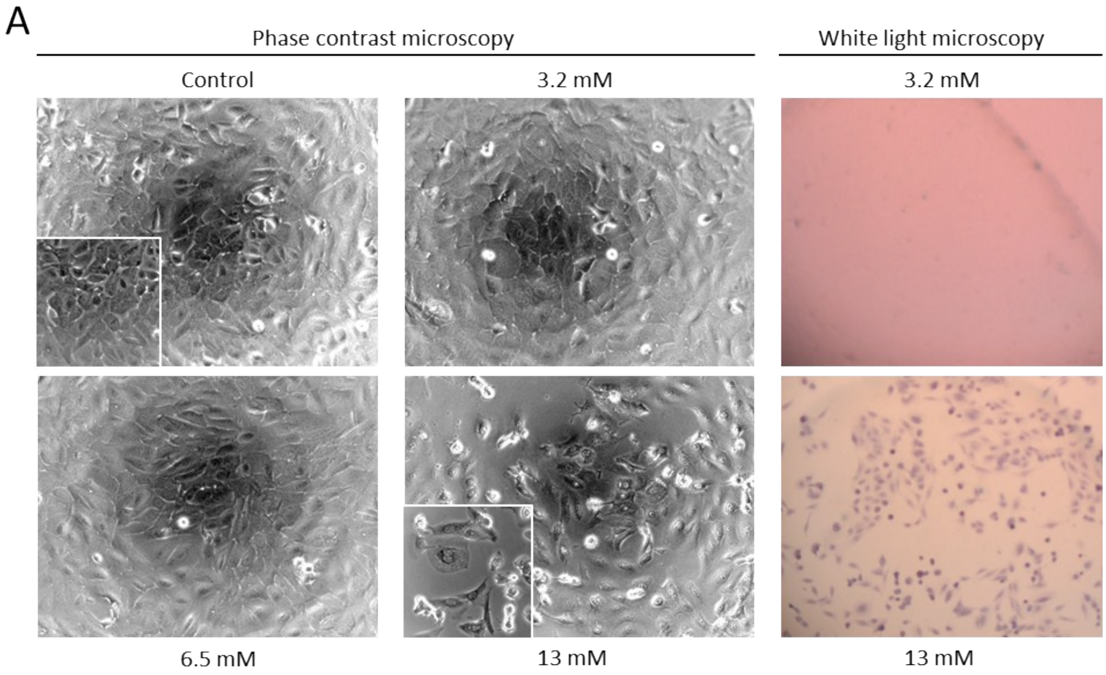

B

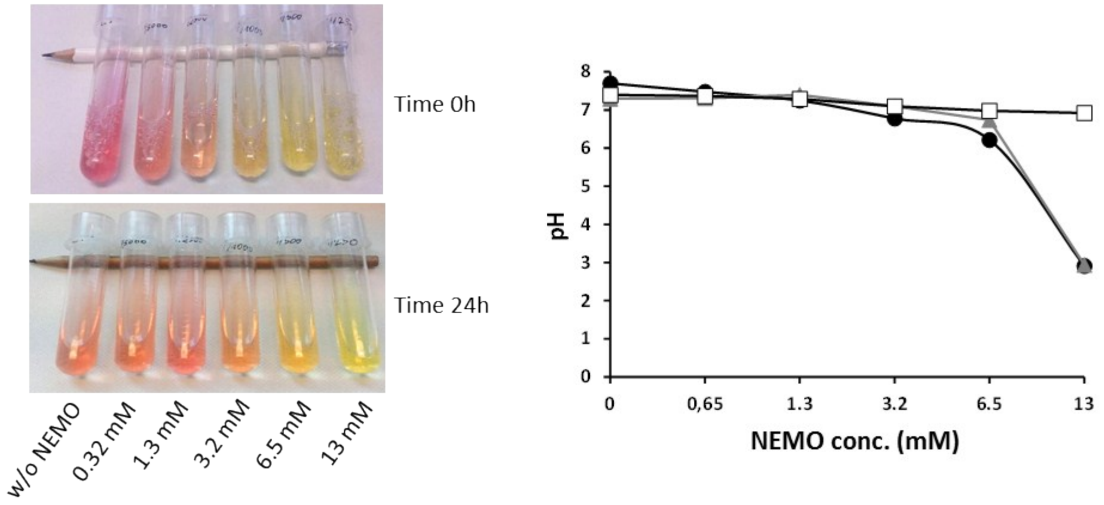

A) Morphology in phase contrast microscopy of progenitor HepaRG cells in untreated cultures (control) and cultures exposed for 24 hours to 3.2, 6.5 and 13mM of methane sulfonic acid:TBD (NEMO) catalyst. While the morphology of cells exposed to 3.2 and 6.5 mM of NEMO catalyst remained similar to that observed in control cells, in cultures of cells incubated with 13mM, the cell density was dramatically reduced, and the cell's cytoplasm appeared granular suggesting a plasma cell membrane disruption. The loss of plasma cell membrane integrity was demonstrated by the staining of cells exposed to 13mM of catalyst with trypan blue, a vital compound that stains the intracellular compartments when plasma cell membrane is damaged or disrupted. In white light microscopy (photographs of the right on panel A), the cells exposed to 3.2 mM of NEMO remained unstained while the cells exposed to 13mM were all strongly positive for the trypan blue staining demonstrating the loss of plasma cell membrane integrity. B) Photographs and C) pH values of culture media without NEMO and with catalyst at concentrations of 0.32, 1.3, 3.2, 6.5 and 13 mM. William's E culture medium contains 10 mg/L of phenol red as pH indicator and is buffered with 2.2 g/L Sodium Bicarbonate. Freshly prepared control medium without incubation in a 5% CO_2 incubator is slightly pink with a pH at ~7.6 (upper panel, Time 0h) while after incubation for 24 hours with 5% CO_2 the control medium in orange and the pH is ~7.2 (lower panel, Time 24h). Addition of low concentrations of NEMO catalyst up to 3.2 did not affect the pH of the culture medium while the highest concentration at 13mM induced a strong acidification with a pH at 2.91. The chart represents the pH values of the media without 5% CO_2 incubation (dark circles), the media with 5% CO_2 incubation (grey triangles) and the media supplemented with 100 mM Hepes (pH 9) with 5% CO_2 incubation (open squares).

2. CHARACTERIZATION DATA

^1H NMR-spectra were recorded using a Bruker AMX-500 apparatus at r.t. in $CDCl_3$ (10mg/0.6ml). Size exclusion chromatography (SEC) was performed in $CHCl_3$ at 30°C using a Triple Detection Polymer Laboratories liquid chromatograph equipped with a refractive index (ERMA 7517), a capillary viscometry, a light scattering RALS (Viscotek T-60) (Polymer Laboratories GPC-RI/CV / RALS) and an automatic injector (Polymer Laboratories GPC-RI/UV) and four columns : a PL gel 10 µm guard column and three PL gel Mixed-B 10 µm columns (linear columns for separation of MWPS ranging from 500 to 10^6 daltons). Differential scanning calorimetry (DSC) measurements were carried out with a DSC Q200 apparatus from T.A. Instruments under nitrogen flow (heating and cooling rate 5 °C /min). Phase contrast microscopy was performed using a Zeiss Axio Vert.A1 inverted microscope while detection of dead cells with trypan blue staining was done with a Zeiss Axioskop microscope. All photographs were analyzed with the Zeiss AxioVision Software. The pH values were measured with a Consort C860 pH meter (Bioblock Scientific).

3. FIGURES

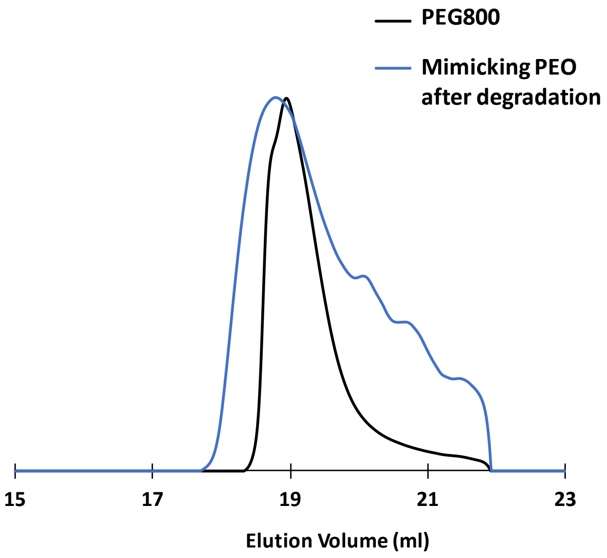

Figure S1. SEC traces overlay of PEG800 macromonomer (black curve) and the mimicking PEG after degradation.
